# Indications for fertility preservation not included in the 2017 Japan Society of Clinical Oncology Guideline for Fertility Preservation in Pediatric, Adolescent, and Young Adult Patients treated with gonadal toxicity, including benign diseases

**DOI:** 10.1007/s10147-021-02082-9

**Published:** 2021-11-17

**Authors:** Masanori Ono, Kimikazu Matsumoto, Narikazu Boku, Nobuharu Fujii, Yumi Tsuchida, Tatsuro Furui, Miyuki Harada, Yoshinobu Kanda, Akira Kawai, Mitsuru Miyachi, Atsuko Murashima, Robert Nakayama, Hiroyuki Nishiyama, Chikako Shimizu, Kazuhiko Sugiyama, Yasushi Takai, Keishi Fujio, Ken-Ichirou Morishige, Yutaka Osuga, Nao Suzuki

**Affiliations:** 1 Department of Obstetrics and Gynecology, Tokyo Medical University, 6-7-1 Nishi-Shinjuku, Shinjuku-ku, Tokyo 160-0023 Japan; 2 Children’s Cancer Center, National Center for Child Health and Development, 2-10-1 Okura, Setagaya-ku, Tokyo 157-8535 Japan; 3 Division of Gastrointestinal Medical Oncology, National Cancer Center Hospital, 5-1-1 Tsukiji, Chuo-ku, Tokyo 104-0045 Japan; 4 Division of Blood Transfusion, Okayama University Hospital, 2-5-1 Shikata-cho, Kita-ku, Okayama-shi, Okayama 700-8558 Japan; 5 Department of Allergy and Rheumatology, Graduate School of Medicine, The University of Tokyo, 7-3-1 Hongo, Bunkyo-ku, Tokyo 113-0033 Japan; 6 Department of Obstetrics and Gynecology, Gifu University Graduate School of Medicine, 1-1 Yanagido, Gifu-shi, Gifu 501-1194 Japan; 7 Department of Obstetrics and Gynecology, Graduate School of Medicine, The University of Tokyo, 7-3-1 Hongo, Bunkyo-ku, Tokyo 113-0033 Japan; 8 Division of Hematology, Department of Medicine, Jichi Medical University, 3311-1 Yakushiji, Shimotsuke-shi, Tochigi 329-0498 Japan; 9 Department of Musculoskeletal Oncology and Rehabilitation Medicine, National Cancer Center Hospital, 5-1-1 Tsukiji, Chuo-ku, Tokyo 104-0045 Japan; 10 Department of Pediatrics, Kyoto Prefectural University of Medicine, 465 Kajii-cho, Kawaramachi-Hirokoji, Kamigyo-ku, Kyoto-shi, Kyoto 602-8566 Japan; 11 Division of Maternal Medicine, Center for Maternal-Fetal, Neonatal and Reproductive Medicine, National Center for Child Health and Development, 2-10-1 Okura, Setagaya-ku, Tokyo 157-8535 Japan; 12 Department of Orthopedic Surgery, Keio University School of Medicine, 35 Shinanomachi, Shinjuku-ku, Tokyo 160-8582 Japan; 13 Department of Urology, Faculty of Medicine and Graduate School of Comprehensive Human Science, University of Tsukuba, 1-1-1 Tennodai, Tsukuba-shi, Ibaraki 305-8577 Japan; 14 Department of Oncology, National Center for Global Health and Medicine Hospital, 1-21-1 Toyama, Shinjuku-ku, Tokyo 162-8655 Japan; 15 Department of Clinical Oncology and Neuro-Oncology Program, Hiroshima University Hospital, 1-2-3 Kasumi, Minami-ku, Hiroshima-shi, Hiroshima 734-8551 Japan; 16 Department of Obstetrics and Gynecology, Saitama Medical Center, Saitama Medical University, 1981 Kamoda, Kawagoe-shi, Saitama 350-8550 Japan; 17 Department of Obstetrics and Gynecology, St. Marianna University School of Medicine, 2-16-1 Sugao, Miyamae-ku, Kawasaki-shi, Kanagawa 216-8511 Japan

**Keywords:** Fertility preservation, Cyclophosphamide, Pediatric, adolescent, and young adults with cancer, Oncofertility, Japan Society of Clinical Oncology, Japan Society for Fertility Preservation

## Abstract

In recent years, local governments in Japan have established a public financial support system for fertility preservation in pediatric, adolescent, and young adult cancer patients. Fertility preservation has become popular for patients with cancers included in the gonadal toxicity risk classification of the 2017 edition of the Guideline for Fertility Preservation in Children, Adolescents and Young Adult Cancer Patients from the Japan Society of Clinical Oncology. However, patients with cancer and non-cancer diseases that are not included in the Guideline’s gonadal toxicity risk classification also often receive treatment that may affect fertility, but they are often denied the opportunity of fertility preservation because no public financial support is available for diseases not listed in the Guideline. The national research project proposes including these diseases in the indications and treatment for fertility preservation. Therefore, we cooperated with the Japan Society for Fertility Preservation and the Ministry of Health, Labour and Welfare research group to solicit opinions from experts in each therapeutic area and reviewed the literature and overseas guidelines. This paper summarizes the findings of the project. We believe that it will be an important source of information for clinicians treating patients who need fertility preservation but note that the appropriateness of fertility preservation for the disorders listed in this report needs to be continuously reviewed as medical care advances.

## Introduction

**Fig. 1:**
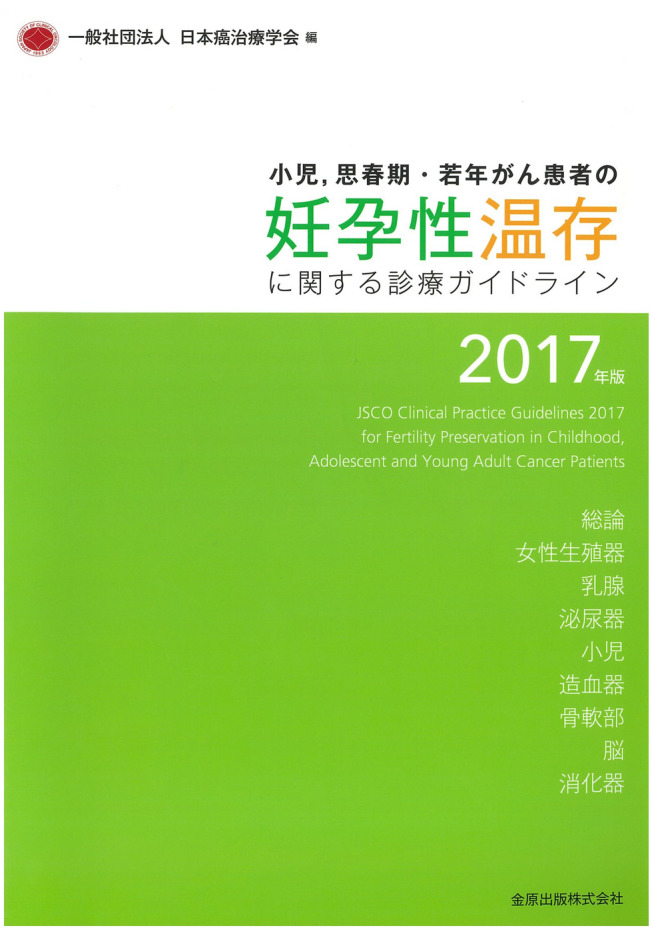
The 2017 edition of the Guideline for Fertility Preservation in Children, Adolescents and Young Adult Cancer Patients by the Japan Society of Clinical Oncology. The gonadal toxicity risk classification in this guideline summarizes malignant diseases for which fertility preservation is indicated

The world's first baby resulting from in vitro fertilization, Louise Brown, was born in Britain in 1978, and in vitro fertilization/embryo transfer technology subsequently started to be used globally as an innovative treatment for infertility. Thereafter, additional technological advancements occurred in the form of embryo freezing and ovarian tissue freezing, and in 2004 Donnez et al. in Belgium reported a spontaneous pregnancy and delivery after ovarian tissue freezing and transplantation in a young patient with stage IV Hodgkin lymphoma [1]. Since the American Society of Clinical Oncology issued a recommendation for fertility preservation in cancer patients in 2006, fertility preservation has been developed to meet the internationally recognized need in young cancer patients [2]. Various organizations have been established in this field, including the US Oncofertility Consortium, the European FertiPROTEKT, the International Society for Fertility Preservation, and the Asian Society for Fertility Preservation. In Japan, the Japan Society for Fertility Preservation (JSFP), the first academic organization specialized in this field, was established in November 2012. The JSFP, together with the Ministry of Health, Labour and Welfare research group, developed a local cooperation network for fertility preservation in cancer patients [3]. In addition, in 2017 the Japan Society of Clinical Oncology issued the Guideline for Fertility Preservation in Children, Adolescents, and Young Adult Cancer Patients (hereafter referred to as the Guideline), which provided guidance for fertility preservation in various types of cancer (Harada et al. submitted, Tozawa et al. submitted). Specifically, this was the first guideline in Japan on clinical questions related to fertility preservation in cancer patients. Moreover, the guideline stressed the need to involve various stakeholders such as nurses, pharmacists, and psychologists and the need for close cooperation between oncologists and reproductive medicine doctors. It also listed indications for fertility preservation (Fig. 1).

However, recommendations for fertility preservation have yet been established for cancers and treatment modalities not listed in the Guideline or for non-cancer disorders and their treatments. Consequently, in such cases healthcare providers in clinical practice (e.g., doctors, nurses, clinical psychologists, pharmacists, social workers) and patients often have to search for information on fertility preservation. On this background and because of the advancements in fertility preservation technology, fertility preservation must be considered for non-cancer disorders and for pediatric, adolescent, and young adult patients with cancer. Moreover, clinicians and patients have an increasing need for information on this topic [4]. Finally, in countries outside Japan, approximately 10% of patients who received fertility preservation were reported to have non-cancer disorders [5, 6]. Making the need for fertility preservation among non-cancer patients clear to healthcare providers will provide opportunities for decision-making based on provision of appropriate counseling and information to patients. Therefore, we aimed to gather information to expand the Guideline to include disorders and treatments not covered in the original version.

## Materials and methods

The national research project in Japan (19EA1015) was initiated in April 2019 and aimed to give all patients the opportunity of fertility preservation. It proposes including additional types of cancer and non-cancer diseases in the indications and treatment for fertility preservation. To determine these indications and treatments, participants in the project cooperated with the JSFP and the Ministry of Health, Labour and Welfare research group to solicit opinions from experts in each therapeutic area and reviewed the literature and overseas guidelines.

## Results

The types of disorder and treatments identified in this study as missing from the Guideline are listed (Table 1), and information is provided that explains why they should be included. The list is arranged according to the affected organ. Note that non-organ-specific diseases requiring hematopoietic stem cell transplantation are included under the section on the hematopoietic system.

**Table 1:** List of disorders and treatments identified in this study as missing from the Guideline for Fertility Preservation in Children, Adolescents and Young Adult Cancer Patients from the Japan Society of Clinical Oncology

Mammary glands	
Hormone therapy (standard duration of therapy: 5–10 years)	
Molecular target therapy (standard duration of therapy: 1 year)	
Urinary organs	
Juvenile bladder cancer (total cystectomy can also affect fertility by causing ejaculation failure)	
Hematopoietic system	
Myelodysplastic syndrome	
Myeloproliferative neoplasm	
Chronic lymphocytic leukemia	
Waldenstrom macroglobulinemia	
Plasma cell neoplasms (multiple myeloma, light chain amyloidosis, polyneuropathy, organomegaly, endocrinopathy, monoclonal gammopathy, and skin changes [POEMS] syndrome)	
Hemophagocytic syndrome	
Chronic active Epstein–Barr virus infection	
Histiocytic and dendritic cell tumors	
Immunodeficiency-related lymphoproliferative tumors	
Autoimmune hemolytic anemia	
Thrombotic thrombocytopenic purpura	
Idiopathic thrombocytopenic purpura	
Pure red cell aplasia	
Pediatric non-cancer disorders	
Aplastic anemia	
Hereditary bone marrow failure syndrome (Fanconi anemia, Diamond Blackfan anemia, congenital keratosis)	
Primary immunodeficiency syndrome	
Inborn errors of metabolism (including adrenoleukodystrophy)	
Osteopetrosis	
Thalassemia	
Sickle cell disease	
Chronic active Epstein–Barr virus infection	
Langerhans cell histiocytosis	
Hemophagocytic lymphohistiocytosis	
Paroxysmal nocturnal hemoglobinuria	
Hypophosphatasia	
Epidermolysis bullosa	
Adrenal spinal cord neuropathy	
Lysosome disease	
Systemic lupus erythematosus	
Lupus nephritis	
Juvenile idiopathic arthritis	
Dermatomyositis	
Sjogren's syndrome	
Nephrotic syndrome	
Vasculitis syndrome	
Acquired hemophilia	
Systemic scleroderma	
Dermatomyositis	
Multiple sclerosis	
Crohn's disease	
Bone soft tissues	
Osteoclastoma	
Desmoid	
Malignant peripheral nerve sheath tumor (of the standard and low-differentiation type)	
Brain	
Malignant lymphoma of the central nervous system	
Digestive system	
Inflammatory bowel disease	
Connective tissue disorders	
Systematic lupus erythematosus	
Alveolar hemorrhage	
Thrombotic microangiopathy	
Polymyositis/dermatomyositis	
Interstitial pneumonia associated with anti-MDA5 antibody-positive dermatomyositis	
Scleroderma	
Vasculitis syndrome	
Nephritis and alveolar hemorrhage	
Polyarteritis nodosa	
Microscopic polyangiitis	
Eosinophilic polyangiitis vasculitis granulomatosis	
Takayasu's arteritis	
Pulmonary arterial hypertension associated with connective tissue disease	
Sjogren's syndrome	
Behcet’s disease	
Lupus nephritis	
Nephrosis syndrome	
Acute progressive nephritis syndrome	

### Mammary glands

Two types of breast cancer treatment that are not included in the Guideline are relevant for fertility preservation: hormone therapy, namely tamoxifen (standard duration of therapy: 5–10 years), and anti-HER2 antibodies, e.g., with pertuzumab, trastuzumab and trastuzumab emtansine (standard duration of therapy: 1 year) [2, 7]. Tamoxifen does not cause gonadal toxicity per se, but, because spontaneous miscarriages, birth defects, and fetal deaths have been reported, the drug should not be administered to pregnant women or women who may be pregnant [8]. Long-term hormone therapy may therefore result in delay of childbearing and expose the patients to a natural age-related decline in ovarian reserve. Likewise, administration of anti-HER2 antibodies to pregnant women should be avoided because of the risk of abortion, intrauterine fetal death, organ dysplasia and oligohydramnios reported in animal studies [9–11]. Although the duration of anti-HER2 therapy is shorter than that of hormone therapy, 1 year can be significant from the perspective of fertility, especially in patients aged 40 years and older. The gonadal toxicities of anti-HER2-targeted therapy have not been elucidated.

### Urinary organs

Bladder cancer treatment can damage the gonads, particularly treatment for juvenile bladder cancer. If patients require chemotherapy, testicular sperm extraction is recommended prior to treatment. Total cystectomy can also affect fertility by causing ejaculation failure, so after treatment, patients will also require testicular sperm extraction for reproduction [12].

### Hematopoietic system

The following diseases may affect the hematopoietic system in young adults: myelodysplastic syndrome, myeloproliferative neoplasm, chronic lymphocytic leukemia, Waldenstrom macroglobulinemia, plasma cell neoplasms (multiple myeloma, light chain amyloidosis, polyneuropathy, organomegaly, endocrinopathy, monoclonal gammopathy, and skin changes [POEMS] syndrome), hemophagocytic syndrome, chronic active Epstein–Barr virus infection, histiocytic and dendritic cell tumors, idiopathic thrombocytopenic purpura, and immunodeficiency-related lymphoproliferative tumors [13]. In Japan, the Research Group on Idiopathic Hematopoietic Disorders issued a clinical guideline for pure red cell aplasia and autoimmune hemolytic anemia, and the Research Group on Blood Coagulation Disorders issued one for thrombotic thrombocytopenic purpura [14].

The diseases in this list may be treated by chemotherapy and hematopoietic stem cell transplantation. Chemotherapy is the first-line treatment, but hematopoietic stem cell transplantation is used in patients with intractable disease. Although many chemotherapy treatments for these diseases are considered to have low risk for gonadal toxicity, regimens containing alkylating agents may affect fertility, in which case fertility preservation should be considered.

### Pediatric non-cancer disorders

The following pediatric non-cancer disorders may be treated by hematopoietic stem cell transplantation if initial treatments are unsuccessful; therefore, patients may require fertility preservation because conditioning regimen for stem cell transplantation has a significant impact on fertility (these diseases are listed by the Ministry of Health, Labor and Welfare in the "Act on Promotion of Appropriate Provision of Hematopoietic Stem Cells Used for Transplantation" in Japan) [15]: aplastic anemia, hereditary bone marrow failure syndrome (Fanconi anemia, Diamond Blackfan anemia, congenital keratosis), primary immunodeficiency syndrome, inborn errors of metabolism (including adrenoleukodystrophy), osteopetrosis, thalassemia, sickle cell disease, chronic active Epstein–Barr virus infection, Langerhans cell histiocytosis, hemophagocytic lymphohistiocytosis, paroxysmal nocturnal hemoglobinuria, hypophosphatasia, epidermolysis bullosa, and lysosome disease.

The effects on fertility vary depending on the conditioning regimen. For example, busulfan and total body irradiation significantly affect fertility [16]. In recent years, some patients have received non-myeloablative conditioning regimen, which may have less influence on fertility than conventional myeloablative conditioning regimen [17]. However, it may involve administration of busulfan. When treating children, adolescents and young adults by hematopoietic stem cell transplantation, clinicians should discuss carefully with the patients and their relatives the ethical aspect of preserving fertility in case of hereditary diseases.

Some pediatric non-cancer disorders are treated with alkylating agents, which can also affect fertility. Such disorders include the following: systemic lupus erythematosus, lupus nephritis, juvenile idiopathic arthritis, dermatomyositis, Sjogren's syndrome, systemic scleroderma, rheumatoid arthritis, multiple sclerosis, Crohn's disease, nephrotic syndrome, vasculitis syndrome, pure red cell aplasia, autoimmune hemolytic anemia, thrombotic thrombocytopenic purpura, and acquired hemophilia. However, in recent years, some of these diseases have been increasingly treated with biologics and less often with alkylating agents. Nevertheless, some patients are still treated with the alkylating agent cyclophosphamide, which affects fertility. US and European guidelines recommend sperm cryopreservation in postpubertal men and fertility preservation with gonadotropin-releasing hormone agonists in postpubertal women [18]. In addition, it is recommended to perform fertility preservation by assisted reproductive technology only during a period when the disease is stable so that the procedure does not exacerbate the disease. Care must also be taken with ovarian stimulation, which involves high estrogen levels and, therefore, may also exacerbate the disease [19]. If an antiphospholipid antibody test is positive, oral aspirin and anticoagulant therapy should be considered [19, 20]. In prepubertal children, depending on the cyclophosphamide dose, fertility preservation may be considered during a period when the disease is inactive, although fertility preservation has not been extensively evaluated in prepubertal children with autoimmune diseases.

### Bone and soft tissues

The bone and soft tissue tumors in which fertility preservation may be relevant include giant cell tumor of bone (GCTB) and desmoid (which have intermediate malignancy).

In recent years, the effectiveness of denosumab therapy has been established for GCTB. Denosumab therapy may be continuously given to pediatric, adolescent, and young adult patients with non-resectable GCTB and may affect fertility [21–23].

In desmoid (desmoid fibromatosis), in which symptoms such as tumor growth and pain can appear and require treatment, antitumor drugs such as methotrexate plus vinblastine combination chemotherapy and the multitarget tyrosine kinase inhibitor pazopanib are increasingly being used in clinical practice and can affect fertility [24–27].

### Brain

A malignant lymphoma of the central nervous system is a typical brain tumor with a relatively young age of onset [28]. Although gonadotropin deficiency is a problem in this type of tumor, fertility preservation is rarely required.

### Digestive system

Treatment for inflammatory bowel disease may affect fertility and require fertility preservation [29, 30].

### Connective tissue disorders

Treatment for various types of connective tissue disorders may affect fertility in young adults, including cyclophosphamide for systematic lupus erythematosus and cyclophosphamide and other alkylating agents for a variety of other disorders [31]. These treatments and disorders are discussed in more detail below.

#### Systemic lupus erythematosus

Cyclophosphamide has been used for many years to treat major organ damage associated with systemic lupus erythematosus [32]. Although the effectiveness of other immunosuppressive drugs is being established in some organ disorders, such as nephritis, cyclophosphamide is still the first-line therapy for organ disorders such as neuropsychiatric lupus [33]. Although evidence shows that cyclophosphamide causes organ damage, it is often used in addition to steroids for alveolar hemorrhage and thrombotic microangiopathy [34, 35]. In many organ disorders, no established protocol has been established for administration of cyclophosphamide, and the dose and frequency of administration are often adjusted according to individual patient characteristics, such as pathological condition, complications, and treatment responsiveness.

#### Polymyositis/dermatomyositis

For interstitial pneumonia associated with polymyositis and dermatomyositis, immunosuppressants (cyclosporine or tacrolimus, azathioprine, and cyclophosphamide) are used in combination with steroids [36]. Interstitial pneumonia associated with anti-MDA5 antibody-positive dermatomyositis is treated with steroids, cyclophosphamide, and calcineurin inhibitor (cyclosporine or tacrolimus) from the early stage [37]. Alkylating agents are less often used for skin and muscle symptoms associated with polymyositis/dermatomyositis, but cyclophosphamide is sometimes used for interstitial pneumonia [36].

#### Scleroderma

In addition to skin sclerosis, scleroderma may involve other organ disorders, such as interstitial pneumonia, pulmonary hypertension, and gastrointestinal lesions. If interstitial pneumonia is progressive or predicted to progress, patients are treated with cyclophosphamide (oral or intermittent high-dose intravenous therapy) or mycophenolate mofetil. In some cases, treatment may be combined with steroids at a moderate or lower dose [38, 39].

#### Vasculitis syndrome

Depending on the type and severity of vasculitis, steroids, immunosuppressants (e.g., cyclophosphamide, methotrexate, azathioprine), or biologics (e.g., rituximab and tocilizumab) may be used in combination [40, 41]. Types of vasculitis in which cyclophosphamide is frequently used include anti-neutrophil cytoplasmic autoantibody-related vasculitis associated with important organ disorders, such as nephritis and alveolar hemorrhage (polyarteritis nodosa, microscopic polyangiitis, and eosinophilic polyangiitis vasculitis granulomatosis) and polyarteritis nodosa associated with important organ disorders, such as gastrointestinal lesions [41, 42]. In addition, cyclophosphamide may be used in other types of vasculitis, such as Takayasu's arteritis, if the patient is refractory other treatments [41].

#### Pulmonary arterial hypertension associated with connective tissue disease

Pulmonary arterial hypertension associated with systemic lupus erythematosus, mixed connective tissue disease, and primary Sjogren's syndrome is treated with moderate or higher doses of steroids and intermittent high-dose cyclophosphamide [43] Depending on the situation, a selective pulmonary vasodilator is used in combination [43]. Connective tissue diseases that are likely to be associated with pulmonary arterial hypertension include systemic lupus erythematosus, mixed connective tissue disease, scleroderma, and primary Sjogren's syndrome. Pulmonary arterial hypertension associated with scleroderma is generally not reported to be responsive to immunosuppressive treatment, but immunosuppressive treatment is reported to be effective for pulmonary arterial hypertension associated with systemic lupus erythematosus, mixed connective tissue disease, and primary Sjogren's syndrome. Steroids and cyclophosphamide may be used for pulmonary arterial hypertension associated with those diseases [43].

#### Behcet’s disease [44, 45]

The following drugs are used, depending on the type and severity of the lesion: steroids (systemic or topical), colchicine, immunosuppressants (e.g., azathioprine, cyclosporine A, cyclophosphamide), biologics (mainly tumor necrosis factor inhibitors).

Glucocorticoids are the main treatment for connective tissue diseases and related disorders, and cyclophosphamide has ample evidence as a concomitant drug, especially in patients with severe life-threatening or organ-threatening diseases. In recent years, immunosuppressants, biologics, and Janus kinase inhibitors have been increasingly used to treat these disorders, and use of alkylating agents (cyclophosphamide) has decreased. However, cyclophosphamide is still used because it is the treatment with most evidence for certain conditions involving damage to important organs. Cyclophosphamide can affect fertility, so cryopreservation of sperm is recommended for men of reproductive age. In women of reproductive age, fertility preservation with gonadotropin-releasing hormone agonists is recommended [19, 20]. Fertility preservation by assisted reproductive technology should be performed during periods when the disease is stable so that the procedure does not exacerbate the disease and care must be taken with the ovarian stimulation because the high estrogen levels may exacerbate the disease [19, 20]. When performing ovarian stimulation, attention should be paid to the risk of thrombosis, especially in the presence of antiphospholipid antibody syndrome [19, 20]. If a test for antiphospholipid antibody is positive, the addition of oral aspirin and/or anticoagulant therapy should be considered [19]. As discussed above, treatment options other than cyclophosphamide have expanded in recent years, and many patients can be treated without using cyclophosphamide. Rheumatologists and obstetricians/gynecologists should cooperate closely with each other and consider the latest information when selecting treatment.

## Discussion

This article summarizes diseases and treatments suitable for fertility preservation that are not listed in the gonadal risk classification of the Guideline. Identifying these diseases and treatments is important to ensure that affected patients have access to fertility preservation measures.

In 2016, Shiga Prefecture launched the first public subsidy program in Japan for fertility preservation for pediatric, adolescent, and young adult cancer patients. In addition, after publication of the Guideline by the Japan Society of Clinical Oncology in 2017, Kyoto Prefecture started the "Kyoto Prefecture Cancer Patient Fertility Preservation Subsidiary System," which requires close cooperation between oncologists and reproductive medicine doctors in accordance with the Guideline, and established a system to prevent patients not being able to receive fertility preservation for financial reasons. Since then, this project has expanded to local governments across the country. In recent years, a regional network of fertility preservation cooperation has been built nationwide that provides relevant information to patients and supports them in decision-making. Nevertheless, the high cost of fertility preservation by assisted reproductive technology that is not covered by health insurance, together with the cost of cancer treatment, can result in some patients not preserving their reproductive functions. Thus, this issue must be urgently addressed.

The relationship between fertility preservation and cancer outcomes must be verified from the perspective of cancer and reproductive medicine, and long-term, close follow-up should be provided by oncologists and reproductive medicine doctors. Therefore, in November 2018, the JSFP established the Japan Oncofertility Registry (JOFR), a registration project that collects data from cancer patients who received fertility preservation counseling or treatment. At present, registration with JOFR is mandatory if patients want to receive subsidies for fertility preservation from the public subsidy system. The purpose of JOFR is to continuously collect useful information on cancer patients with fertility problems with the aim to help better understand the current status of cancer and reproductive medicine (fertility preservation counseling and fertility preservation) in Japan and treatment outcomes (e.g., cancer treatment outcomes; presence or absence of children; and clinical course of pregnancy/delivery).

Patients requiring fertility preservation who were included in the ongoing survey by the JOFR do not have only malignant diseases, such as cancer, but also blood disorders requiring hematopoietic stem cell transplantation and autoimmune diseases not listed in the Guideline. International fertility preservation guidelines are also not limited to cancer but classify gonadal toxicity by treatment method [46, 47]. Clarifying all the diseases and treatments that require fertility preservation in pediatric, adolescent, and young adult patients will contribute to the advancement of fertility preservation care.
