# Correction to: Indications for fertility preservation not included in the 2017 Japan Society of Clinical Oncology Guideline for Fertility Preservation in Pediatric, Adolescent, and Young Adult Patients treated with gonadal toxicity, including benign diseases

**DOI:** 10.1007/s10147-022-02138-4

**Published:** 2022-02-21

**Authors:** Masanori Ono, Kimikazu Matsumoto, Narikazu Boku, Nobuharu Fujii, Yumi Tsuchida, Tatsuro Furui, Miyuki Harada, Yoshinobu Kanda, Akira Kawai, Mitsuru Miyachi, Atsuko Murashima, Robert Nakayama, Hiroyuki Nishiyama, Chikako Shimizu, Kazuhiko Sugiyama, Yasushi Takai, Keishi Fujio, Ken-Ichirou Morishige, Yutaka Osuga, Nao Suzuki

**Affiliations:** 1 Department of Obstetrics and Gynecology, Tokyo Medical University, 6-7-1 Nishi-Shinjuku, Shinjuku-ku, Tokyo 160-0023 Japan; 2 Children’s Cancer Center, National Center for Child Health and Development, 2-10-1 Okura, Setagaya-ku, Tokyo 157-8535 Japan; 3 Division of Gastrointestinal Medical Oncology, National Cancer Center Hospital, 5-1-1 Tsukiji, Chuo-ku, Tokyo 104-0045 Japan; 4 Division of Blood Transfusion, Okayama University Hospital, 2-5-1 Shikata-cho, Kita-ku, Okayama-shi, Okayama 700-8558 Japan; 5 Department of Allergy and Rheumatology, Graduate School of Medicine, The University of Tokyo, 7-3-1 Hongo, Bunkyo-ku, Tokyo 113-0033 Japan; 6 Department of Obstetrics and Gynecology, Gifu University Graduate School of Medicine, 1-1 Yanagido, Gifu-shi, Gifu 501-1194 Japan; 7 Department of Obstetrics and Gynecology, Graduate School of Medicine, The University of Tokyo, 7-3-1 Hongo, Bunkyo-ku, Tokyo 113-0033 Japan; 8 Division of Hematology, Department of Medicine, Jichi Medical University, 3311-1 Yakushiji, Shimotsuke-shi, Tochigi 329-0498 Japan; 9 Department of Musculoskeletal Oncology and Rehabilitation Medicine, National Cancer Center Hospital, 5-1-1 Tsukiji, Chuo-ku, Tokyo 104-0045 Japan; 10 Department of Pediatrics, Kyoto Prefectural University of Medicine, 465 Kajii-cho, Kawaramachi-Hirokoji, Kamigyo-ku, Kyoto-shi, Kyoto 602-8566 Japan; 11 Division of Maternal Medicine, Center for Maternal-Fetal, Neonatal and Reproductive Medicine, National Center for Child Health and Development, 2-10-1 Okura, Setagaya-ku, Tokyo 157-8535 Japan; 12 Department of Orthopedic Surgery, Keio University School of Medicine, 35 Shinanomachi, Shinjuku-ku, Tokyo 160-8582 Japan; 13 Department of Urology, Faculty of Medicine and Graduate School of Comprehensive Human Science, University of Tsukuba, 1-1-1 Tennodai, Tsukuba-shi, Ibaraki 305-8577 Japan; 14 Department of Oncology, National Center for Global Health and Medicine Hospital, 1-21-1 Toyama, Shinjuku-ku, Tokyo 162-8655 Japan; 15 Department of Clinical Oncology and Neuro-Oncology Program, Hiroshima University Hospital, 1-2-3 Kasumi, Minami-ku, Hiroshima-shi, Hiroshima 734-8551 Japan; 16 Department of Obstetrics and Gynecology, Saitama Medical Center, Saitama Medical University, 1981 Kamoda, Kawagoe-shi, Saitama 350-8550 Japan; 17 Department of Obstetrics and Gynecology, St. Marianna University School of Medicine, 2-16-1 Sugao, Miyamae-ku, Kawasaki-shi, Kanagawa 216-8511 Japan

## Correction to: International Journal of Clinical Oncology (2022) 27:301–309 10.1007/s10147-021-02082-9

The article, Indications for fertility preservation not included in the 2017 Japan Society of Clinical Oncology Guideline for Fertility Preservation in Pediatric, Adolescent, and Young Adult Patients treated with gonadal toxicity, including benign diseases written by Masanori Ono, Kimikazu Matsumoto, Narikazu Boku, Nobuharu Fujii, Yumi Tsuchida, Tatsuro Furui, Miyuki Harada, Yoshinobu Kanda, Akira Kawai, Mitsuru Miyachi, Atsuko Murashima, Robert Nakayama, Hiroyuki Nishiyama, Chikako Shimizu, Kazuhiko Sugiyama, Yasushi Takai, Keishi Fujio, Ken‑Ichirou Morishige, Yutaka Osuga, and Nao Suzuki was originally published Online First without Open Access.

With the author(s)’ decision to opt for Open Choice the copyright of the article changed on February 16, 2022 to © Author(s) 2021 and the article is forthwith distributed under a Creative Commons Attribution 4.0 International License, which permits use, sharing, adaptation, distribution and reproduction in any medium or format, as long as you give appropriate credit to the original author(s) and the source, provide a link to the Creative Commons licence, and indicate if changes were made. The images or other third party material in this article are included in the article’s Creative Commons licence, unless indicated otherwise in a credit line to the material. If material is not included in the article’s Creative Commons licence and your intended use is not permitted by statutory regulation or exceeds the permitted use, you will need to obtain permission directly from the copyright holder. To view a copy of this licence, visit http://creativecommons.org/licenses/by/4.0.

The original article has been corrected.

